# A Genome-Wide Assay Specifies Only GreA as a Transcription Fidelity Factor in *Escherichia coli*

**DOI:** 10.1534/g3.118.200209

**Published:** 2018-05-16

**Authors:** Charles C. Traverse, Howard Ochman

**Affiliations:** Department of Integrative Biology, University of Texas, Austin, Texas 78712

**Keywords:** transcription errors, mutations, fidelity factors, *E. coli*, *greA*

## Abstract

Although mutations are the basis for adaptation and heritable genetic change, transient errors occur during transcription at rates that are orders of magnitude higher than the mutation rate. High rates of transcription errors can be detrimental by causing the production of erroneous proteins that need to be degraded. Two transcription fidelity factors, GreA and GreB, have previously been reported to stimulate the removal of errors that occur during transcription, and a third fidelity factor, DksA, is thought to decrease the error rate through an unknown mechanism. Because the majority of transcription-error assays of these fidelity factors were performed *in vitro* and on individual genes, we measured the *in vivo* transcriptome-wide error rates in all possible combinations of mutants of the three fidelity factors. This method expands measurements of these fidelity factors to the full spectrum of errors across the entire genome. Our assay shows that GreB and DksA have no significant effect on transcription error rates, and that GreA only influences the transcription error rate by reducing G-to-A errors.

All organisms are subject to non-heritable errors that are introduced into RNA during transcription. Although these errors are transient, they contribute considerable variation to the proteome and in the modification of proteins sequences; and in humans, these errors have been associated with aging and the development of cancer (Brégeon and Doetsch 2011). In bacteria, transcription errors occur approximately 10,000-fold more frequently than mutations in DNA and are prevalent across the entire transcriptome (Springgate and Loeb 1975; Rosenberger and Foskett 1981; Rosenberger and Hilton 1983; Imashimizu *et al.* 2013; Traverse and Ochman 2016). It has been estimated that about 1 in 10 proteins would be altered due to the high rate of transcription errors (Traverse and Ochman 2016). Although these transient errors have been hypothesized to have some benefit under stressful conditions (D’Ari and Casadesús 1998; Gordon *et al.* 2009, 2015; Meyerovich *et al.* 2010), most are probably deleterious and generate harmful or non-functional protein variants that need to be degraded. 

In addition to variant proteins that originate from transcription errors, misincorporations can stall RNAP to interfere with DNA replication (Trautinger *et al.* 2005; Tehranchi *et al.* 2010; Dutta *et al.* 2011; Washburn and Gottesman 2011; Zhang *et al.* 2014; Gamba *et al.* 2017). When an error occurs during transcription, the misincorporated base triggers the RNAP to halt transcription and translocate backward along the DNA template while simultaneously extruding the error from the RNAP, a process called “backtracking” (Komissarova and Kashlev 1997; Nudler *et al.* 1997). If this backtracked RNAP is not resolved, RNAPs can accumulate upstream, posing a barrier to DNA replication enzymes and generating double-strand breaks (Trautinger *et al.* 2005; Tehranchi *et al.* 2010; Dutta *et al.* 2011; Washburn and Gottesman 2011; Zhang *et al.* 2014). To mitigate the effects of transcription errors, bacteria have evolved quality-control strategies that serve to restart backtracked RNAP: the RNAP can either undergo intrinsic cleavage, whereby the RNAP itself catalyzes the removal of the misincorporated base (Orlova *et al.* 1995; Zenkin *et al.* 2006; Yuzenkova and Zenkin 2010; Mishanina *et al.* 2017), or the error can be removed by Gre-mediated cleavage, in which secondary proteins bind to the RNAP and induce transcript cleavage (Borukhov *et al.* 1993, 2001; Laptenko *et al.* 2003).

Two Gre proteins, GreA and GreB, restart paused RNAPs by resolving backtracked RNAP and, as a result, resolve errors that prompted the RNAP to pause. These proteins are considered to be transcription fidelity factors (or anti-backtracking factors) since they have been shown to remove misincorporations in *in vitro* transcription assays and *in vivo* reporter gene assays (Feng *et al.* 1994; Toulmé *et al.* 2000; Laptenko *et al.* 2003; Gordon *et al.* 2013). Recently, a sequencing-based study recognized a role for GreAB in reducing G→A errors (James *et al.* 2016); however, that methodology is prone to sequencing artifacts, even after strict quality control. Additionally, that sequencing study measured the nascent transcripts that reside within paused RNAP, some of which may not have undergone intrinsic or Gre-mediated cleavage. Consequently, the effects of GreAB on the rates and profiles of errors that are incorporated into the transcriptome remain unexplored. 

Recently, DksA, which competes for the same binding site as GreA and GreB on the RNAP, has been identified as a third transcription fidelity factor based on *in vivo* and *in vitro* assays (Roghanian *et al.* 2015; Satory *et al.* 2015). DksA, which is structurally similar to GreA and GreB, does not induce transcript cleavage but instead reduces the occurrence of transcription errors through an unidentified mechanism (Zenkin and Yuzenkova 2015). Moreover, the error rate and the types of errors prevented by DksA remain unknown. In this study, we employ a technique that eliminates sequencing artifacts (Acevedo *et al.* 2014; Acevedo and Andino 2014), and has allowed us to advance the measurement of transcription error rates to all types of substitutions, including base substitutions and indels, across the entire transcriptome. Our assay found no effect of GreB and DksA on the transcription error rate, and that GreA reduces only the rate of G→A errors, as previously reported (James *et al.* 2016). These results suggest that intrinsic cleavage, although slow, may have a larger role in resolving misincorporated bases than previously expected.

## Materials And Methods

### Bacterial strains and growth conditions

All strains used in this study were derivatives of *Escherichia coli* MG1655. Mutant strains harboring deletions of *greA*, *greB* or *dksA* were supplied by M. Cashel (NIH), and new strain constructs harboring deletions in one, two, or three of these genes were generated with P1*vir*, as described previously (Miller 1972). Bacteria were grown in LB to facilitate growth, avoid auxotrophies of the mutant strains, and because it has been shown that there are no differences in the transcription error rate when compared to growth in chemically defined minimal media (Traverse and Ochman 2016). Cultures and plates were supplemented with antibiotics as appropriate: chloramphenicol (Cm: 20 µg/ml), kanamycin (Kan: 40 µg/ml), and tetracycline (Tet: 20 µg/ml). 

### RNA extractions

For RNA extractions, newly transduced strains (to avoid the accumulation of suppressor mutations) were grown without antibiotics, and RNA was extracted during log-phase growth. RNA was isolated using the RNA*snap* protocol for gram-negative bacteria, as previously described (Stead *et al.* 2012; Traverse and Ochman 2016). Ribosomal RNAs were removed from the total RNA preparations using the MICROBExpress kit (Life Technologies), according to manufacturer’s instructions. Each sample represents an independent biological replicate that originated from independent cultures. 

### Library preparation and sequencing

The CirSeq method for preparing and sequencing RNA libraries was performed as described in Acevedo *et al.* (2014), with minor modifications (Traverse and Ochman 2016). Purified mRNA was mechanically sheared to 80–100 nt fragments, which were then fractionated and extracted by urea-PAGE. Isolated mRNAs were circularized, primed with random hexamers, and reverse transcribed, resulting in linked repeats of each original mRNA fragment. Resulting cDNAs were sheared into fragments 300–450 bp in length, and libraries prepared using the NEBNext Ultra RNA Library Prep Kit for Illumina sequencing (NEB). Samples were barcoded and sequenced on a MiSeq v3 platform generating 300-bp reads.

### Data Analysis

Sequences were processed using the CirSeq_v3 pipeline (Acevedo *et al.* 2014) to generate the consensus among the cDNA repeats within a sequencing read using default settings and a quality score cutoff of 20. Subsequent analyses were performed with the same custom python scripts previously described for the analysis of base substitutions (Traverse and Ochman 2016) and transcription indels (Traverse and Ochman 2017). The overall error rate was calculated by dividing the total number of transcription errors by the total number of bases sequenced in the transcriptome. For individual error rates, the total number of errors for each error type was divided by the total number of bases sequenced in the transcriptome, such that the sum of all individual error rates is equal to the overall error rate. Additionally, the individual error rates were normalized by base composition, as previously described (Traverse and Ochman 2016). The error rates associated with nucleotides preceding a particular focal error were normalized by the nucleotide composition of positions -1 to -7 relative to each of the four bases. This was accomplished by randomly sampling the sequenced transcriptome one million times each for A, C, G, and T as the focal nucleotide and calculating the base composition for the eight bases preceding each sampled focal nucleotide. All statistics were performed in Prism Graphpad or R. 

### Data availability

The sequences can be found online in NCBI with the BioProject Accession PRJNA417942. Transcription errors can be found in Table S1. Supplemental material available at Figshare: https://doi.org/10.6084/m9.figshare.6275990.

## Results

**Figure 1:**
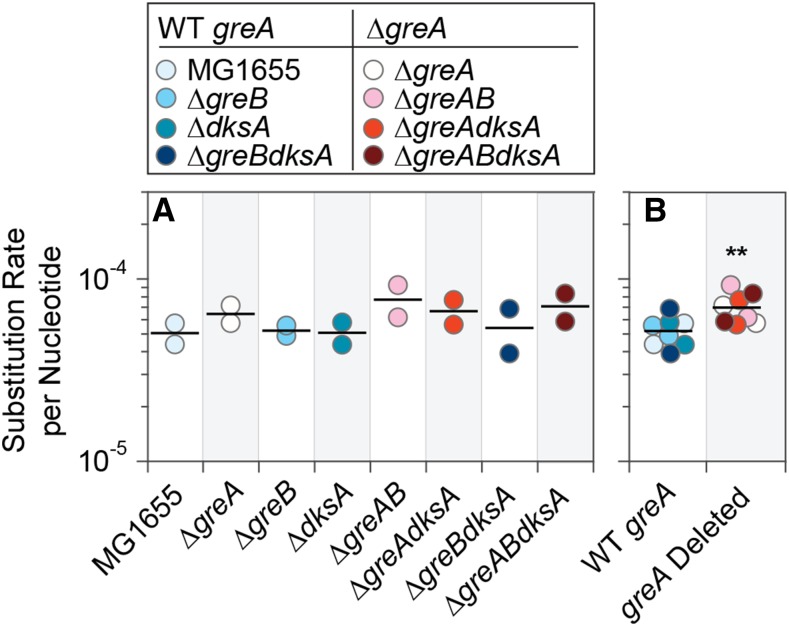
Transcription error rates in *E. coli* strains lacking one or multiple fidelity factors. A. Rates of transcription base substitutions in wildtype *E. coli* strain MG1655 and in isogenic strains harboring deletions of all possible combinations of three fidelity factors, *greA*, *greB*, and *dksA*. There are no significant differences of the transcription substitutions rates between wild-type *E. coli* MG1655 and any the fidelity factor mutants (unpaired Student’s *t*-tests, n *=* 2, *P* > 0.2). B. Rates of transcription substitutions of all strains with an intact *greA* gene (blue-shaded points) and all mutants lacking the *greA* gene (red-shaded points). The overall error rate in Δ*greA* strains is significantly higher than in strains with wild-type *greA* (Mann-Whitney *U*-test, **P = 0.01). The same y-axis is used as in A.

To determine the effects of GreA, GreB, and DksA on transcriptional fidelity, we used a transcriptome-wide sequencing approach that discriminates sequencing artifacts from actual errors that arose during transcription by circularizing mRNAs, reverse-transcribing the circularized fragments, and sequencing cDNAs that contain multiple linked repeats of the original mRNA fragment (Acevedo *et al.* 2014; Acevedo and Andino 2014). A consensus sequence is then calculated from the repeats to recognize errors arising during library preparation and sequencing (which only occur once per repeat) from errors that were present in the original mRNA fragment (which appear in every repeat). Applying this method to measure the transcription error rate in mutant strains lacking one, or any of the possible combinations, of these genes (including the triple mutant), yielded no mutant strains that differed significantly from one another or from the wildtype (Figure 1A; unpaired Student’s *t*-tests, n *=* 2, *P* > 0.2). However, there was a tendency for mutants lacking the *greA* gene (*i.e.*, Δ*greA*, Δ*greAgreB*, Δ*greAdksA*, Δ*greAgreBdksA*; red-shaded points in Figure 1) to have slightly higher error rates than strains that possessed an intact *greA* gene, even in combination with a deletion in one or both of the other fidelity factors (*i.e.*, MG1655, Δ*greB*, Δ*dksA*, Δ*greBdksA*; blue-shaded points in Figure 1). By grouping strains based on their possession or lack of *greA*, the transcription base substitutions rate was significantly higher in Δ*greA* strains (Figure 1b, Mann-Whitney *U*-test, n *=* 8, *P =* 0.007), indicating that GreB and DksA do not contribute to overall transcriptional fidelity under the conditions tested (Table S1).

**Figure 2:**
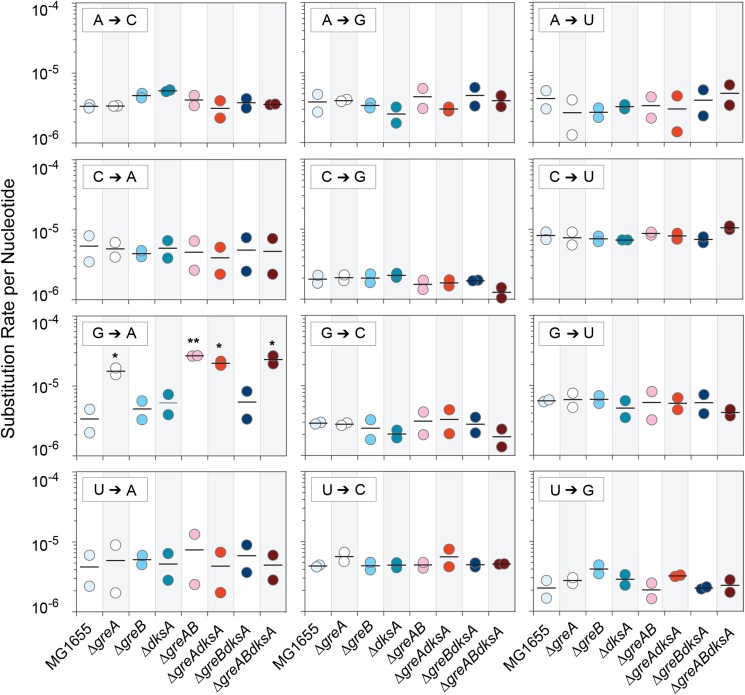
Transcription error rate for each type of base substitution in wild-type *E. coli* MG1655 and each fidelity factor mutant. Each of the mutant strains with *greA* deleted have a significantly higher G→A substitution rate than wild-type *E. coli* MG1655 (unpaired Student’s *t*-tests: Δ*greA*, *P =* 0.027; Δ*greAgreB*, *P =* 0.003; Δ*greAdksA*, *P =* 0.011; Δ*greAgreBdksA*, *P =* 0.027). No other comparisons were statistically significant. All tests were subject to correction for multiple tests by the Benjamini-Hochberg procedure with a false discovery rate of 0.05. *P = 0.05; **P = 0.01.

We next sought to determine if specific base substitutions were differentially affected by each of the transcription fidelity factors. In those mutant strains that harbored an intact *greA* (Δ*greB*, Δ*dksA*, Δ*greBdksA*), there were no significant effects on the error rates of individual substitutions (Figure 2); however, G→A substitutions were significantly higher in all Δ*greA* strains (Figure 2). This trend remains when all statistical tests were performed on strains grouped according to whether or not they possessed an intact *greA* gene, an intact *greB* genes, or an intact *dksA* gene (Supplementary Fig S1 *P =* 0.0001, Mann-Whitney *U*-test corrected by Benjamini-Hochberg procedure with a false discovery rate of 0.05).

**Figure 3:**
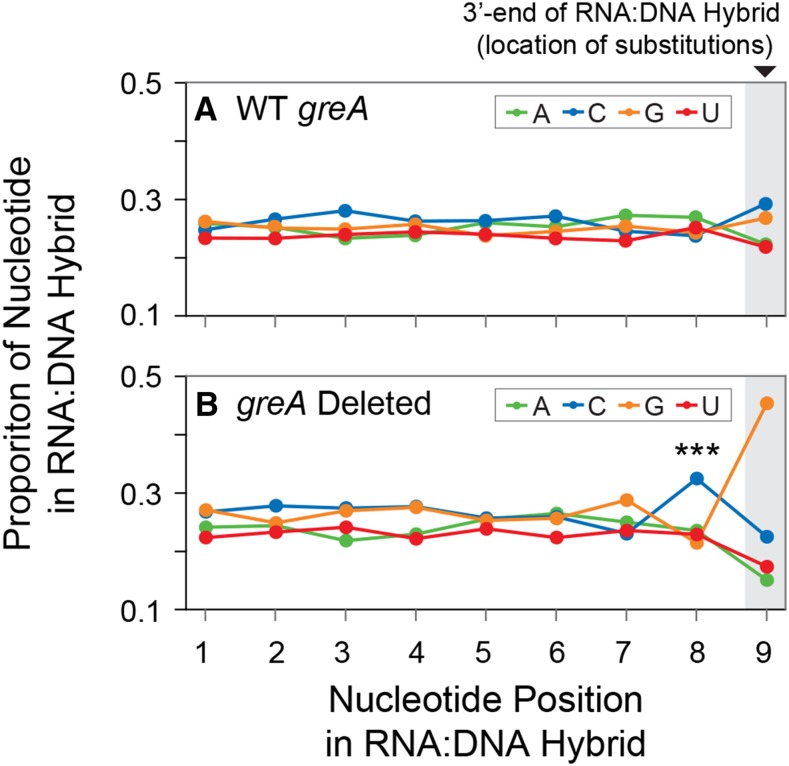
Nucleotide composition in the RNA:DNA hybrid at positions preceding a transcription error. The proportion of each nucleotide at each position within the RNA:DNA hybrid was calculated for all strains with an intact wild-type *greA* gene and in which the *greA* gene was deleted. The shaded gray area marks the 3′-end of the RNA:DNA hybrid at the site where the transcription error occurred. In strains lacking *greA*, the occurrence of C was significantly higher in the position immediately before a transcription error (Fisher’s exact test, *** P = 0.0001), and no other positions in the RNA:DNA hybrid exhibit a significant difference in nucleotide composition between strains. The results for each position were normalized by the base composition of the sequenced transcriptome. All tests were subject to correction for multiple tests by the Benjamini-Hochberg procedure with a false discovery rate of 0.05.

**Figure 4:**
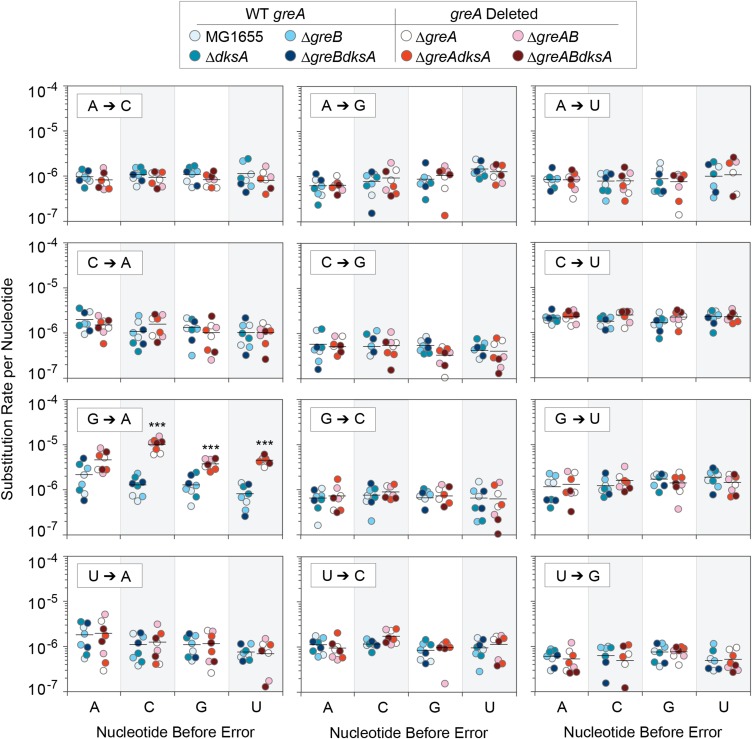
Effect of preceding nucleotide on error rates of each substitution type. The transcription error rate was calculated for all replicates with wild-type *greA* and all Δ*greA* replicates when each nucleotide occurs before each substitution type. The G→A substitution rate is significantly higher for Δ*greA* replicates when preceded by C, G, and T (Mann-Whitney tests, n *=* 8, *P =* 0.0002 when preceded by C, G, and T). All tests were subject to correction for multiple tests by the Benjamini-Hochberg procedure with a false discovery rate of 0.05. ***P = 0.001.

During transcription, the nine most recently transcribed bases remain hybridized to the template DNA within the RNAP (known as the RNA:DNA hybrid), and previous work has suggested that these bases may influence the error rate (James *et al.* 2016). To determine if the most recently transcribed RNA influences the error rate, we analyzed the occurrence of each of the four nucleotides in bioinformatically reconstructed RNA:DNA hybrids (Traverse and Ochman 2017) immediately preceding each of the observed errors. We found that cytosine was significantly overrepresented in the position immediately preceding a transcription error in Δ*greA* mutant strains (Figure 3). We examined this in further detail by analyzing how each of the four nucleotides influenced the error rate for each substitution type (Figure 4). We found that G→A substitutions were significantly more likely to occur if any nucleotide but A preceded the substitution in the Δ*greA* mutant, with the strongest effect produced by C. No other nucleotide preceding any of the other type of base substitution significantly increased or decreased the error rate.

Our analysis focused on errors that resulted in base substitutions, but transcription errors can also produce insertions or deletions. None of the mutant strains, or groupings of strains, displayed a significant effect on transcription indel rates (Table S1), nor did they cause differences in errors according to the strand or genomic location of transcription, or the level of gene expression. 

## Discussion

Three transcription fidelity factors—GreA, GreB, and DksA—have been described in *E. coli* (Feng *et al.* 1994; Toulmé *et al.* 2000; Laptenko *et al.* 2003), and by applying a transcriptome-wide approach that registers all errors suppressed by these factors (Acevedo *et al.* 2014; Acevedo and Andino 2014), we conclude that, under the conditions tested, GreB and DksA do not significantly influence transcriptional fidelity and that GreA reduces only the G→A error rate. Indeed, a recent study used circle sequencing to demonstrate that the *Saccharomyces cerevisiae* TFIIS gene, the eukaryotic homolog of GreA, reduces the G→A error rate more than all other errors (Gout *et al.* 2017), indicating that the preponderance of G→A errors in mutants lacking fidelity factors may be universal. Our finding that the other recognized fidelity factors are of little consequence in correcting transcription errors counters previous views on GreB-mediated cleavage. Prior work has suggested that GreB increases transcription fidelity *in vitro* (Erie *et al.* 1993); however, further support for the action of GreB on transcription fidelity has been extrapolated either from its ability to cleave backtracked transcripts (Toulmé *et al.* 2000; Fish and Kane 2002; Borukhov *et al.* 2005; Zenkin and Yuzenkova 2015) or from studies that test Δ*greAB* mutants and cannot disentangle the individual contributions of the two proteins (Imashimizu *et al.* 2013; James *et al.* 2016). 

Recently, information on RNAP pausing from an alternate transcriptome-wide approach, termed NET-seq (Churchman and Weissman 2011; Larson *et al.* 2014), was used to examine the effect of Δ*greAB* mutants on rates of transcript misincorporation (James *et al.* 2016). NET-seq captures transcript sequences that reside within the RNAP (*i.e.*, before most error correction can occur) and yields error rates that are orders of magnitudes higher than we obtained when surveying transcripts that have been released from the RNAP. The difference between these rates is that the estimates obtained through NET-seq can include errors that have not yet undergone intrinsic cleavage as well as those in transcripts that are eventually aborted and are not part of the mature transcriptome. In line with our results, only the G→A error rate substantially increased in the Δ*greAB* mutant when assayed by NET-seq, although it was not determined if the effect was attributable solely to GreA (James *et al.* 2016). 

We also found evidence of biases in bases preceding certain errors. NET-seq found the C was more likely to be transcribed prior to a G→A error and we found similar results with CirSeq: C had the largest effect on the G→A error rate, but G and T were also elevated prior to G→A errors. The mechanism underlying the increase of C nucleotides immediately preceding a G→A error is unclear from our results. For example, using our methodology, it is not possible to determine if all errors increase subsequent to transcription of cytosine but intrinsic cleavage is able to correct all errors except for G→A, or if only G→A errors are increased following cytosine. It is possible that the 3′-nt structure of A (misincorporated opposite of C) influences either the intrinsic cleavage of the misincorporated nucleotide or the ability of the RNAP to detect the misincorporation event. However, previous *in vitro* work does not indicate that G→A is harder to resolve through intrinsic cleavage than N→A errors (Zenkin *et al.* 2006), but these measurement did not take into account all possible preceding nucleotides.

If NET-seq only registered transcripts prior to error correction, it would yield the same error rates for wild-type and Δ*greAB* mutants, due to the fact that Gre acts on transcripts after misincorporation. That the G→A error rate increases in Δ*greAB* mutants relative to wild-type indicates that NET-seq interrogates not only those transcripts that never experienced an error and those that have not undergone intrinsic or Gre-mediated cleavage, but also those that have already undergone intrinsic or Gre-mediated cleavage (Imashimizu *et al.* 2015; James *et al.* 2016). A previous study concerning *Thermus aquaticus* RNAP has shown that intrinsic cleavage mechanisms remove misincorporations involving adenine at much faster rates than other misincorporations (Zenkin *et al.* 2006), and consequently, the actual input of G→A errors is likely higher than the 10-fold increase reported for the Δ*greAB* mutant assayed by NET-seq. Although G→A errors should be removed by intrinsic cleavage at a faster rate than other errors (Zenkin *et al.* 2006), it appears that the input of these errors is so high that it requires the additional action of Gre-mediated cleavage. It is important to note that intrinsic cleavage has been measured *in vitro* as being very slow, and consequently, intrinsic cleavage was not thought to significantly contribute to transcription fidelity. However, the low error rates that we obtained suggest that intrinsic cleavage may operate at a faster rate *in vivo* or that there is possibly an as-yet unidentified cleavage factor.

The NET-seq findings support our results, but they only assayed a double mutant and did not separate the individual effects of GreA and GreB. We find that GreB does not act on any class of transcription errors, which is inconsistent with prior findings (Erie *et al.* 1993) and views (Toulmé *et al.* 2000; Fish and Kane 2002; Borukhov *et al.* 2005; Zenkin and Yuzenkova 2015) on GreB-mediated cleavage. However, a recent study that used an *in vivo* reporter system to specifically probe G→A errors reported that GreA, and not GreB, affected the G→A error rate (Bubunenko *et al.* 2017), but that overexpressing GreB in the Δ*greA* mutant could mitigate G→A errors. Because GreB operates on transcription errors only under atypical conditions (*i.e.*, at very high concentrations in strains lacking *greA*) suggests that GreA is the major fidelity factor and implies that GreB has a separate function (Feng *et al.* 1994; Toulmé *et al.* 2000; Bubunenko *et al.* 2017). 

The difference between the results obtained for GreA and GreB can be traced to their roles in inducing cleavage in RNAPs that have backtracked by different lengths: GreA preferentially associates with backtracks of only 2 or 3 bases, whereas GreB associates with backtracks up to 18 bases in length (Borukhov *et al.* 1992, 2005; Feng *et al.* 1994; Hsu *et al.* 1995; Toulmé *et al.* 2000). And because most misincorporations that occur during transcription induce short backtracking events (Sosunov *et al.* 2003; Zenkin *et al.* 2006; Mishanina *et al.* 2017), GreA will be the dominant, if not sole, fidelity factor detected by *in vivo* systems. GreA and GreB were originally classified as transcription fidelity factors due to their ability to induce nucleolytic cleavage of misincorporated transcripts; however, they also serve as anti-backtracking factors that prevent DNAP-RNAP collisions (Trautinger *et al.* 2005; Tehranchi *et al.* 2010; Dutta *et al.* 2011). Therefore, GreA may not increase fidelity *per se* but instead may restart backtracked RNAP, such that increased fidelity is a consequence of restarting transcription. 

The third fidelity factor tested was DksA, which is known to have a role in transcription initiation (Paul *et al.* 2004, 2005; Perederina *et al.* 2004; Potrykus *et al.* 2006), elongation (Zhang *et al.* 2014), and genome stability (Trautinger *et al.* 2005; Tehranchi *et al.* 2010; Dutta *et al.* 2011). DksA and Gre have similar structures and RNAP binding locations, but unlike Gre, DksA does not induce nucleolytic cleavage (Vinella *et al.* 2012). Whereas a study showed that DksA reduces transcript read-through by inhibiting misincorporations *in vitro* and *in vivo* (Roghanian *et al.* 2015), this error avoidance mechanism is not observed in our assay. Additionally, a Δ*dksA* mutant increases the readout of transcription errors in a reporter assay (Satory *et al.* 2015); however, transcription errors were not measured directly such that error rates could not be derived. The discrepancies between our transcriptome-wide analyses and these assay systems suggest a subtle role for this protein that possibly occurs below our limit of detection or under conditions not tested, such as during amino acid starvation (where ppGpp could act synergistically with DksA; (Vinella *et al.* 2012; Roghanian *et al.* 2015)) or the general stress response (Dutta *et al.* 2011; Zhang *et al.* 2014). Under such conditions, transcription and translation can become uncoupled, and when RNAP and the ribosome do not physically interact, RNAP is prone to pausing (Zhang *et al.* 2014). Although misincorporations induce RNAP pausing (James *et al.* 2016; Gamba *et al.* 2017) and this pausing is known to be mitigated by DksA (Zhang *et al.* 2014), the degree to which this protein helps prevent errors across the transcriptome is not yet evident.

Therefore, of the three previously identified fidelity factors, only GreA appears to act as a fidelity factor. Because we only tested the roles of GreA, GreB, and DksA under a single condition, it is important to note that they could possibly affect transcription fidelity under other assay conditions (*e.g.*, stationary phase, stringent response, general stress response, etc.). Furthermore, the ∼100-fold difference between our reported G→A error rates in Δ*greA* mutants and those reported in Bubunenko *et al.* (2017) may stem from the different assay conditions: if the reporter-based assay induces stressful conditions, then the fidelity factors may become more important for error correction than in the conditions used in our study. Alternatively, this difference may stem from error rates that occur below our limit of detection. Although GreB and DksA may serve roles outside of error correction, our findings indicate that neither GreB nor DksA significantly influences transcription fidelity, as was found previously for GreB (Bubunenko *et al.* 2017). Additionally, intrinsic cleavage is considered a slow and inefficient mechanism of transcription error correction; however, we suggest that it may emend the majority of transcription misincorporations with additional action of GreA to remove G→A errors. Recent evidence shows that Gre-mediate cleavage inhibits DNA break repair (Sivaramakrishnan *et al.* 2017), perhaps explaining why we find that only G→A errors are corrected by external factors.
